# Long-term neurodevelopmental outcomes should be addressed in robot-assisted abdominal surgery in small infants

**DOI:** 10.1097/JS9.0000000000002366

**Published:** 2025-03-28

**Authors:** Yun Zhang, Ting Gong, Xiaojuan Zhang

**Affiliations:** a Children’s Hospital Affiliated to Zhengzhou University, Henan Children’s Hospital, Zhengzhou Children’s Hospital, Zhengzhou, China; b Chongqing college of Traditional Chinese Medicine, Chongqing, People’s Republic of China; c Chongqing Medical and Pharmaceutical College, Chongqing, China

*Dear Editor,*

The study by Jin *et al* evaluated the feasibility and safety of robot-assisted abdominal surgery in infants under 5 months of age, comparing outcomes with laparoscopic-assisted procedures^[^1^]^. The authors provided valuable insights into the technical applicability of the Da Vinci system in this vulnerable population. However, further discussion is required about critical methodological limitations and long-term developmental outcomes.

The retrospective design introduces inherent biases, particularly selection bias, as surgical modality was determined by surgeon preference and parental choice rather than randomization. The small cohort size, especially for intestinal duplication (*n* =7 robotic vs. *n* =9 laparoscopic), limits statistical power and generalizability. Furthermore, the absence of standardized protocols for postoperative care or follow-up introduces variability in outcome assessments. While statistical analyses (e.g., Mann-Whitney *U* test) were appropriately applied, the lack of adjustment for confounding variables, such as preoperative comorbidities or surgeon experience, weakens causal inferences.

The study demonstrated that robot-assisted surgery reduced intraoperative bleeding in Hirschsprung disease cases, albeit with longer operative times and higher costs. Although there were no significant differences in complications or short-term recovery metrics (e.g., fasting time, hospital stay), but robotic procedures requiring extended operative times in two of three disease groups. Prolonged anesthesia exposure in infants was associated with potential neurocognitive risks^[^2,3^].^ The authors did not address critical neurodevelopmental considerations. Additionally, the impact of pneumoperitoneum duration and intraoperative hemodynamic changes on cerebral perfusion in infants remains unexplored.

Younger and lower body weight at surgery and anesthesia exposure could be associated with negatively influence cognitive and behavioral development^[^4^]^. Future studies should incorporate longitudinal neurodevelopmental assessments (e.g., Bayley Scales of Infant Development) and neuroimaging to evaluate structural and functional outcomes.

Jin *et al* provide preliminary evidence supporting the technical feasibility of robot-assisted surgery in infants. However, the study’s retrospective design, small sample size, and lack of long-term data preclude definitive conclusions about safety and broader applicability. To advance this field, multicenter prospective trials with rigorous neurodevelopmental follow-up are essential. As robotic platforms evolve, pediatric-specific innovations, such as miniaturized instruments and reduced setup times, may mitigate current limitations and enhance safety in this fragile population.

## Data Availability

Not applicable.
